# Cognitive tutoring induces widespread neuroplasticity and remediates brain function in children with mathematical learning disabilities

**DOI:** 10.1038/ncomms9453

**Published:** 2015-09-30

**Authors:** Teresa Iuculano, Miriam Rosenberg-Lee, Jennifer Richardson, Caitlin Tenison, Lynn Fuchs, Kaustubh Supekar, Vinod Menon

**Affiliations:** 1 Department of Psychiatry and Behavioral Sciences, Stanford University, Stanford, California 94305, USA; 2 Department of Special Education, Vanderbilt University, Nashville, Tennessee 37203, USA; 3 Department of Neurology and Neurological Sciences, Stanford University, Stanford, California 94305, USA; 4 Stanford Neurosciences Institute, Stanford, California 94305, USA

## Abstract

Competency with numbers is essential in today's society; yet, up to 20% of children exhibit moderate to severe mathematical learning disabilities (MLD). Behavioural intervention can be effective, but the neurobiological mechanisms underlying successful intervention are unknown. Here we demonstrate that eight weeks of 1:1 cognitive tutoring not only remediates poor performance in children with MLD, but also induces widespread changes in brain activity. Neuroplasticity manifests as normalization of aberrant functional responses in a distributed network of parietal, prefrontal and ventral temporal–occipital areas that support successful numerical problem solving, and is correlated with performance gains. Remarkably, machine learning algorithms show that brain activity patterns in children with MLD are significantly discriminable from neurotypical peers before, but not after, tutoring, suggesting that behavioural gains are not due to compensatory mechanisms. Our study identifies functional brain mechanisms underlying effective intervention in children with MLD and provides novel metrics for assessing response to intervention.

## Abstract (web summary)

Mathematical learning disabilities (MLD) affect up to 20% of children and are linked to poorer socioeconomic and health outcomes in adulthood. Here, Iuculano *et al*. show that tutoring induced functional changes in multiple brain systems underlie remediation of poor maths skills in children with MLD.

## Graphical abstract

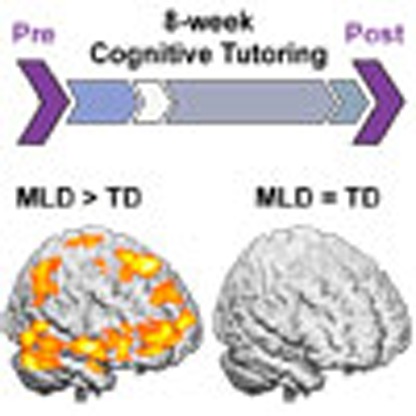

The use of mathematics to categorize, visualize and manipulate information extends to virtually all domains of human activity in the modern world^1^,^2^,^3^,^4^,^5^,^6^. Yet, mathematical difficulties are widespread in school-age children, adolescents and even college students^1^,^7^,^8^,^9^,^10^,^11^, and up to 20% of individuals have some form of mathematical learning disability (MLD)^1^,^5^,^11^,^12^. Individuals with MLD have specific difficulties with numerical and arithmetic problem solving, despite age-appropriate schooling and absence of impairments in other cognitive domains^1^,^13^. Although the prevalence rate of MLD is comparable, or even higher, to that of reading disabilities^14^, MLD has received considerably less attention from the research community^1^. This is surprising because, to an even greater extent than reading skills, mathematical abilities in early childhood have been shown to impact later academic and professional achievement^15^, socioeconomic well being^5^,^16^ and health outcomes^2^,^3^,^4^. It is therefore imperative to increase mathematical competence in all children, but most importantly in those falling at the lower end of the distribution^1^,^14^.

School-based interventions designed to strengthen mathematical problem solving skills have been shown to be effective in improving performance in some children with MLD^17^,^18^,^19^,^20^,^21^, but the neurobiological consequences of these programs are still unknown^1^. Although lesion studies of individuals with math impairments have primarily focused on the specific role of the parietal cortex, recent neuroimaging research has begun to suggest that MLD involves aberrations in multiple functional systems^1^,^6^,^22^,^23^,^24^. These include brain systems implicated in visual form judgement and symbol recognition, anchored in the ventral temporal–occipital cortex; quantity and magnitude processing, anchored in the intraparietal sulcus region of the parietal cortex; as well as attention and working memory functions, supported by a frontoparietal control network^1^,^6^,^22^,^23^,^24^. However, the profile of differences in brain activation is currently an unresolved issue, as previous research has reported both over^25^,^26^,^27^,^28^,^29^,^30^ and underactivation^31^,^32^,^33^ in MLD, relative to control groups. This lack of consensus is likely due to the limited number of studies, the use of different cutoff criteria for identifying MLD groups, inadequate matching on general cognitive measures such as intelligence quotient (IQ), reading and working memory, as well as the use of wide age ranges, and diverse experimental tasks and control conditions. Despite the lack of consistency in the direction of brain activations, previous studies have hinted that individuals with MLD show atypical functional responses in multiple brain areas, and not just parietal regions involved in quantity manipulation^25^,^27^,^28^,^29^.

The extent to which effective behavioral intervention can alter aberrant activations in distributed brain systems is currently unknown. Specifically, it is not known whether successful behavioural interventions can effectively normalize activity in the same systems that show aberrant functional responses in MLD, or whether children with MLD recruit atypical neural resources to achieve the same level of performance as typically developing (TD) children. A comprehensive quantitative characterization of functional brain changes following effective intervention is required to address these questions and to elucidate the mechanisms by which poor math problem-solving skills can be remediated in children with MLD. This, in turn, can help identify neural factors mediating individual differences in response to intervention.

Importantly, as with other learning disabilities^34^, response to interventions varies considerably across individuals^35^. Analyses of individual differences have pointed to behavioural factors, such as severity of symptoms at the beginning of treatment (that is, severity of math disability), or domain-general cognitive abilities as potential mediators of success or failure in response to population-wise effective interventions^34^,^36^,^37^. Here we investigate the possibility that poor response to intervention may be associated with weak functional neuroplasticity in children with MLD^38^.

We combined cognitive assessments with event-related functional magnetic resonance imaging (fMRI) acquired during arithmetic problem solving (Fig. 1a), and advanced multivariate pattern classification analyses, to investigate performance and brain changes associated with an intensive 8-week 1:1 math tutoring program^17^,^18^,^19^,^39^,^40^,^41^. We used a tutoring program that combines conceptual aspects of number knowledge and speeded practice on efficient counting strategies and systematic learning of number families. These components are designed to facilitate arithmetic fluency, and have previously been validated in school settings^17^,^18^,^19^,^39^,^40^,^41^ (Fig. 1a,b).

We studied a well-characterized group of 7–9-year olds with MLD (Supplementary Tables 1 and 2), and a group of TD children matched on age, gender, IQ, reading and working memory abilities (Supplementary Table 1). First, we use voxel-wise univariate fMRI analysis to identify brain systems that are aberrant in children with MLD during numerical problem solving, and to characterize over or underactivation profiles of such aberrations, relative to TD children. Second, by combining univariate analyses with multivariate techniques of brain patterns classification, we directly test three competing hypotheses of functional brain changes in MLD: the (i) neural normalization hypothesis, which posits that atypical brain responses in children with MLD before tutoring become more similar to, and statistically indistinguishable from TD peers after tutoring; (ii) persistent neural aberration hypothesis, which posits that, even if performance deficits are successfully remediated, children with MLD will continue to show atypical responses in the same brain areas that they did before tutoring; and (iii) neural compensation hypothesis, which posits that children with MLD, after tutoring, would recruit additional and distinct (compensatory) brain systems compared with TD children. Finally, we developed novel machine-learning-based quantitative metrics to investigate whether a systematic relation exists between tutoring-induced neuroplasticity and behavioural gains in children with MLD.

We find that, consistent with the multi-component neurocognitive model of MLD^6^,^22^,^23^,^24^,^42^, before tutoring, the profile of brain responses in children with MLD during arithmetic problem solving is characterized by prominent and widespread aberrations in multiple functional systems serving quantity, visuospatial, attention, working memory and cognitive control processes, that support successful numerical problem solving^1^,^6^,^22^. Critically, 8 weeks of 1:1 tutoring results in marked changes in brain responses across all these systems. These widespread changes are characterized by normalization of responses, such that brain activation patterns in MLD children are no longer discriminable from their TD peers after tutoring. Crucially, multivariate pattern analyses provide novel evidence that the degree of such widespread tutoring-induced functional brain plasticity is uniquely associated with individual differences in response to intervention in children with MLD.

## Results

To investigate behavioural and neurobiological consequences of 8 weeks of 1:1 math tutoring, we examined differences in performance and task-based fMRI activation levels during arithmetic problem solving, between and within groups, before and after tutoring (see Methods). Because tutoring could also alter performance and brain function in TD children, tutoring-induced changes in the MLD group were assessed with respect to both (a) pre-tutoring and (b) post-tutoring sessions in the TD control group.

### Tutoring normalizes performance in children with MLD

**Figure 1:**
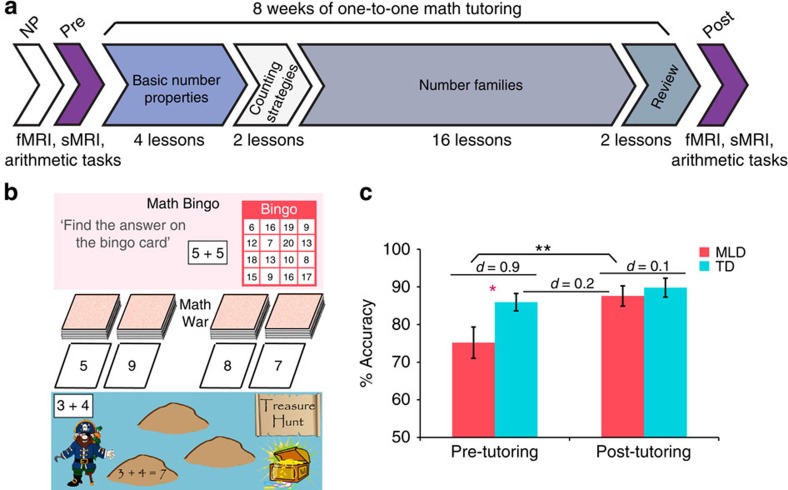
Overall study design, math games and behavioural results. (**a**) Before 8 weeks of tutoring, all children underwent an extensive battery of neuropsychological (NP) assessments for IQ, academic achievement, and working memory. Before tutoring, each child underwent a functional MRI (fMRI) scan session during which they had to verify addition equations—Addition task (that is, 3+4=7), and assess the validity of number identity expressions—Control task (that is, 7=7). High-resolution structural MRI (sMRI) images were also acquired in each participant for anatomical co-registration. During this session, and before entering the scanner, children also performed an arithmetic production task (that is, 4+3=?). On successful completion of the aforementioned sessions, children went through an intensive 8-week, 1:1 tutoring program focused on conceptual aspects of number knowledge and speeded practice on efficient counting strategies and systematic learning of number families (that is, all the problems that summed to 5, and the corresponding subtraction problems). After 8 weeks of tutoring all children underwent a second MRI scan session. (**b**) Examples of the physical math games used in the tutoring: Math Bingo—in which the child has to calculate the sum of a given problem and verify whether the answer is on their Bingo card; Math War—in which the child competes with the tutor to get the highest sum from their decks of cards; and *Treasure Hunt*—in which the child has to calculate the answer of a given problem, and write down both the equation and its correct solution on the stepping stones of the ‘treasure map' to get to the treasure chest. (**c**) Performance normalization on the arithmetic problem-solving task in children with MLD (*n*=15) after 8 weeks of math tutoring, plotted against TD (*n*=15) children's performance at pre- and post- tutoring sessions. Error bars indicate one s.e.m. *P<0.05; **P<0.01, significant by independent samples *t*-test. Effect sizes for group differences are shown as Cohen's *d*.

We first examined changes in math performance following 8 weeks of tutoring and, as predicted by evidence from previous school-based studies^17^,^19^,^40^, we found that this type of 1:1 tutoring is effective in children with MLD, as assessed by significant improvements in accuracy on the arithmetic problem solving verification task performed inside the scanner (*t*_(14)_=3.323, *P*=0.005, Cohen's *d*=0.86) (Fig. 1c). Moreover, we found evidence for performance normalization in MLD: before tutoring, children with MLD were significantly less accurate than their TD peers (*t*_(28)_=−2.318, *P*=0.028, Cohen's *d*=0.85), while their accuracy performance after tutoring did not differ from TD children at pre-tutoring (*t*_(28)_=0.471, *P*=0.64, Cohen's *d*=0.17), or post-tutoring (*t*_(28)_=−0.598, *P*=0.55, Cohen's *d*=0.22; Fig. 1c). The TD group did not show significant accuracy gains after tutoring (*t*_(14)_=1.469, *P*=0.16, Cohen's *d*=0.37). However, they showed significantly reduced reaction times after tutoring (*t*_(14)_=−4.951, *P*=0.0001, Cohen's *d*=1.29), while this reduction was not evident in the MLD group (*t*_(14)_=−1.188, *P*=0.25, Cohen's *d*=0.33). The groups did not differ on reaction times before (*t*_(28)_=−0.694, *P*=0.49, Cohen's *d*=0.25) or after (*t*_(28)_=1.267, *P*=0.22, Cohen's *d*=0.46) tutoring.

Critically, these results were replicated in a separate arithmetic problem-solving task performed outside the scanner in which, instead of verifying addition equations, children were asked to verbally generate the answer to addition problems (Methods). Here again, performance differences that were evident between MLD and TD groups before tutoring (*t*_(25)_=−2.631, *P*=0.014, Cohen's *d*=1.01), were entirely absent after tutoring (*t*_(25)_=−1.141, *P*=0.26, Cohen's *d*=0.44; *t*_(25)_=0.007, *P*=0.99, Cohen's *d*=0.01 for TD's performance before and after tutoring, respectively; Supplementary Fig. 1). These data demonstrate that tutoring-induced performance normalization cannot be explained by confounding factors related to being in the scanner the second time such as greater familiarity and/or diminished anxiety, and furthermore, it is independent of the response mode and the format in which the problems are presented.

### Tutoring normalizes brain activity in children with MLD

**Figure 2:**
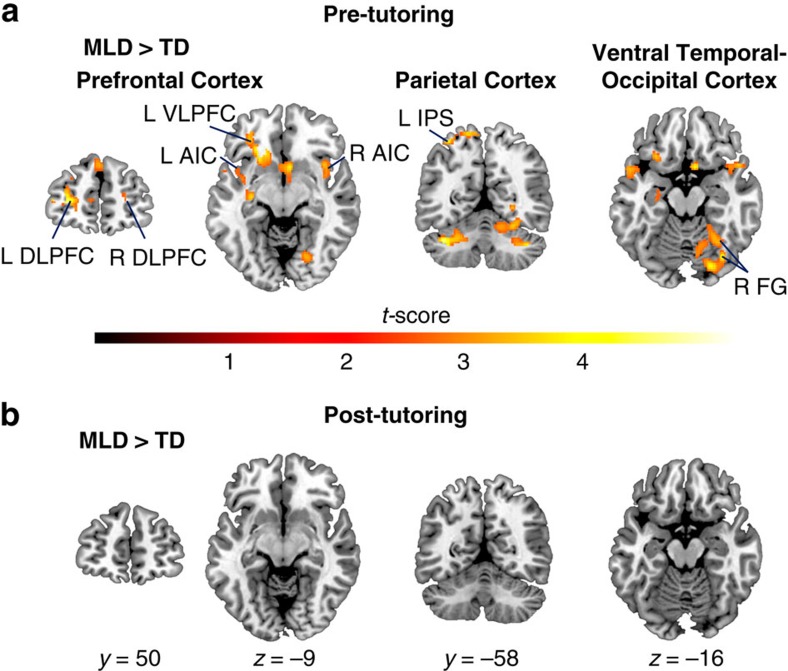
Normalization of aberrant functional brain responses in children with MLD after 8 weeks of math tutoring. (**a**) Before tutoring, children with MLD (*n*=15) showed significant differences in brain activation levels compared with TD children (*n*=15). Significant group differences were evident in multiple cortical areas in the Prefrontal Cortex, including the bilateral Dorsolateral Prefrontal Cortices (DLPFC), and the left Ventrolateral Prefrontal Cortex (VLPFC), as well as the bilateral Anterior Insular Cortices (AIC); in the Parietal Cortex encompassing the left Intraparietal Sulcus (IPS); and in the Ventral Temporal–Occipital Cortex including the right Fusiform Gyrus (FG). (**b**) After 8 weeks of tutoring, functional brain responses in MLD children (*n*=15) normalized to the levels of TD children (*n*=15). Height threshold *P*<0.01, extent threshold *P*<0.01, significant by whole-brain voxel-wise independent samples *t*-test.

Before tutoring, relative to TD children, children with MLD showed higher activation levels in multiple brain systems. Specifically, they showed higher activity in the bilateral prefrontal cortices including the dorsolateral and ventrolateral prefrontal cortices, the bilateral anterior insular cortices (Fig. 2a, Supplementary Table 3), the bilateral superior frontal gyri, and the right orbitofrontal cortex (Supplementary Table 3). Higher activation levels in the MLD group, compared to TD peers, were also seen in the inferior and superior parietal cortices encompassing the left intraparietal sulcus (Fig. 2a, Supplementary Table 3), the left supramarginal gyrus, and the right precuneus (Supplementary Table 3), as well as in the ventral temporal–occipital cortex including the bilateral fusiform gyri and the right lingual gyrus (Fig. 2a, Supplementary Table 3). Subcortical areas, including the bilateral cerebellum, right subcallosal cortex, left putamen and left anterior hippocampus and adjoining amygdaloid complex, also showed significantly higher functional activation in the MLD group compared with the TD group before tutoring (Supplementary Table 3). Additional analysis controlling for accuracy scores before tutoring identified similar profiles of overactivation in the prefrontal, parietal, and ventral temporal–occipital cortices (Supplementary Fig. 2). This result suggests that differences in brain activation cannot be explained by performance differences between the groups before tutoring.

Before tutoring, TD children also showed significant activation in similar prefrontal, parietal and ventral temporal–occipital cortical areas as the MLD group, but their functional responses were much more focal (Supplementary Fig. 3, Supplementary Table 4). Crucially, compared with children with MLD, they did not show significantly higher activation levels in any brain regions. To summarize these findings, before tutoring, group differences in brain responses to arithmetic problems were characterized by overengagement of distributed brain regions across prefrontal, parietal and ventral temporal–occipital cortices important for numerical problem solving^6^,^43^,^44^, and previously reported to be aberrantly engaged in MLD^25^,^27^,^28^,^29^.

To assess the nature of tutoring-induced functional brain changes in children with MLD we then examined brain responses in regions that were overengaged by MLD children before tutoring. After 8 weeks of tutoring, children with MLD did not show greater activity in any of these brain regions when compared with either (a) pre-tutoring or (b) post-tutoring sessions in TD children (Fig. 2b), consistent with the neural normalization hypothesis, rather than the persistent neural aberration hypothesis. To further validate these findings we used Bayesian estimation analyses, which provide complete distributions of credible values for group means and their differences^45^. These analyses were conducted on beta parameter estimates from the individual General Linear Model (GLM) analysis for all brain areas that displayed significant differences between MLD and TD children before tutoring (Supplementary Table 3). We found that the differences in mean betas between the groups were non-significant in the post-tutoring data (all *P*>0.15), with the 95% highest density confidence intervals all centered on 0 (Supplementary Figs 4 and 5). This result suggests that the lack of differences in functional brain activation between MLD and TD children during the post-tutoring session cannot be explained by low statistical power.

To test the neural compensation hypothesis, we examined whether tutoring elicited the recruitment of additional brain regions in the MLD group, when compared with the TD group. Notably, after tutoring, children with MLD did not engage any other brain area more than their TD peers at either (a) pre-tutoring or (b) post-tutoring sessions (Fig. 2b). Similarly, after tutoring, TD children did not show greater activation levels in any of the brain systems undergoing functional normalization in the MLD group. However, after tutoring, compared with children with MLD, TD children showed higher activation levels in the right ventrolateral prefrontal cortex, and in the left motor cortex (Supplementary Fig. 6, Supplementary Table 5). An additional analysis of group (MLD, TD) by tutoring-session (pre, post) interactions identified the same brain systems described above (Supplementary Fig. 7, Supplementary Table 6).

**Figure 3:**
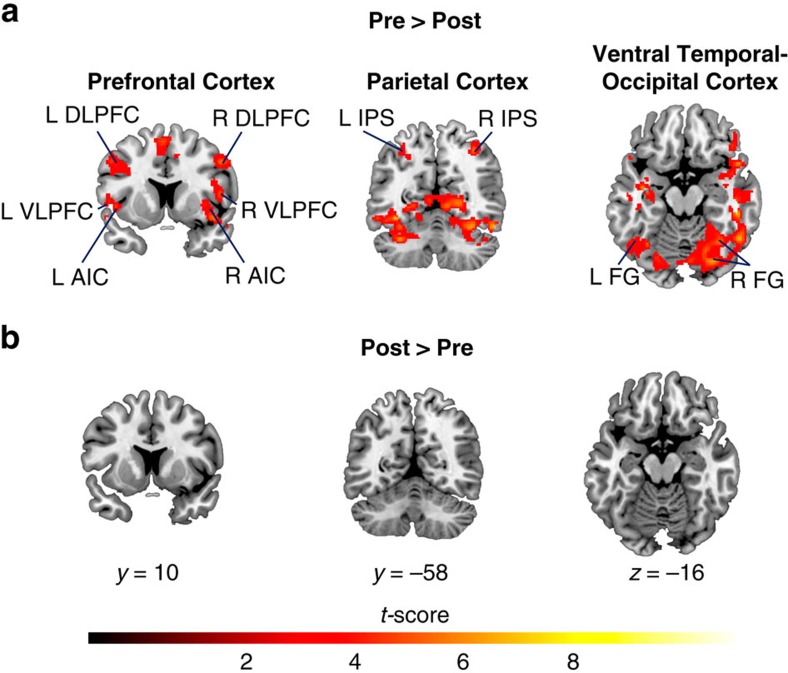
Tutoring-induced functional brain plasticity in children with MLD. (**a**) Before tutoring, compared with post-tutoring, children with MLD (*n*=15) exhibited overactivation in several cortical areas in the Prefrontal Cortex, including the bilateral Dorsolateral and Ventrolateral Prefrontal Cortices (DLPFC and VLPFC), and the bilateral Anterior Insular Cortices (AIC); in the Parietal Cortex, encompassing the bilateral Intraparietal Sulci (IPS); and in the Ventral Temporal–Occipital Cortices, including the bilateral Fusiform Gyri (FG). (**b**) Post-tutoring, compared to pre-tutoring, no brain areas showed greater activation in children with MLD (*n*=15). Height threshold *P*<0.01, extent threshold *P*<0.01, significant by whole-brain voxel-wise paired-samples *t*-test.

To further characterize the nature of tutoring-induced functional brain plasticity in children with MLD, we conducted additional within-group analyses contrasting brain activity levels before and after tutoring. Compared with the post-tutoring session, functional activation before tutoring was characterized by greater signal intensity levels in the same brain systems described above, encompassing prefrontal, parietal and ventral temporal–occipital cortices (Fig. 3a, Supplementary Table 7). Crucially, children with MLD did not show greater functional activation in any of these or other brain regions after tutoring (Fig. 3b).

Taken together, these results provide convergent evidence for the hypothesis that 1:1 math tutoring can normalize activity levels in children with MLD, to the level of their TD peers, in multiple brain systems important for numerical problem solving that are aberrant in children with MLD^6^,^22^,^23^,^24^,^42^.

### Normalization of brain activity patterns in MLD

**Figure 4:**
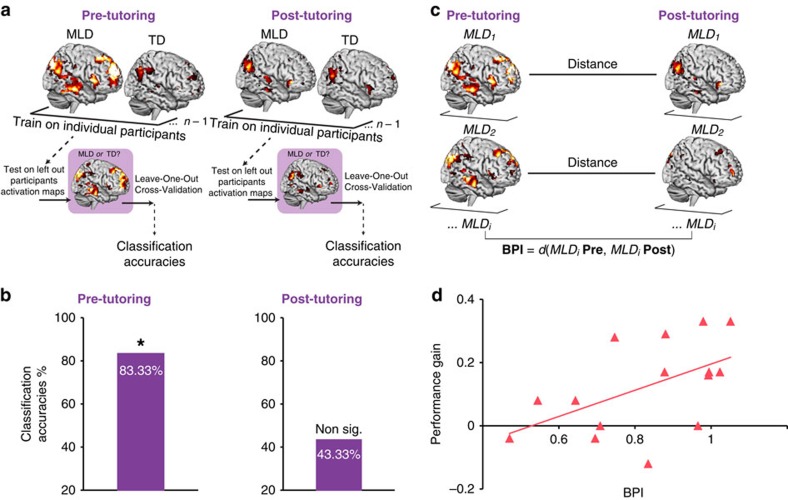
Multivariate brain activity patterns-based classification of MLD children and association with performance gains. (**a**) Classification Analysis flowchart. A linear classifier built using support vector machines (SVM) with Leave-One-Out Cross-Validation (LOOCV) was used to classify children with MLD (*n*=15) from TD (*n*=15) children based on patterns of brain activation during arithmetic problem solving, before and after tutoring. (**b**) Classification accuracies pre- and post-tutoring. Brain activation patterns between MLD (*n*=15) and TD (*n*=15) children during arithmetic problem solving were significantly and highly discriminable before tutoring at 83.33% accuracy (*P*=0.01), while the groups were no longer discriminable by their patterns of brain activity after tutoring (43.33%, *P*=0.42). (**c**) Brain Plasticity Index (BPI) in children with MLD. A distance metric *d* was computed to quantify tutoring-induced functional brain plasticity effects pre-tutoring versus post-tutoring in children with MLD (*n*=15). *d* was calculated individually for each MLD child by computing a multivariate spatial correlation between pre- and post-tutoring patterns of brain activity, and subtracting it from 1. (**d**) Relation between tutoring-induced functional brain plasticity and performance gain. A significant positive correlation (*r*=0.526; *P*<0.05) was observed between BPI and individual performance gains associated with tutoring in children with MLD (*n*=15). Performance gain represents change in arithmetic problem solving accuracy from pre- to post-tutoring.

To further evaluate the neural normalization hypothesis, and contrast it with the persistent neural aberration hypothesis in a quantitatively rigorous manner, we used Support Vector Machine (SVM)^46^, a multivariate classification technique (Methods), to assess whether functional brain activity patterns in the brain systems identified above (Fig. 2a) could be used, over and above signal level intensity differences, to discriminate children with MLD from their TD peers either before or after tutoring (Fig. 4a). Brain activity patterns (*t*-maps) were first *z*-transformed (demeaned and scaled by variance), thus ensuring that group differences are independent of differences in task-related activity levels. The *z*-transformed activity maps were used as input features to a pattern-based SVM classifier^46^, and Leave-One-Out Cross-Validation (LOOCV) procedures were used to assess discriminability of task-related activation patterns between the MLD and TD groups. We hypothesized that if, before tutoring, and in line with our univariate analysis, children with MLD engage brain areas differently, their task-related brain activity patterns would be highly discriminable from the TD group. Consistent with this hypothesis, multivariate pattern analyses revealed high (83.33%), and significant (*P*=0.01), classification accuracies in discriminating MLD from TD children before tutoring (Fig. 4b). This classification analysis was repeated using post-tutoring brain activity patterns as the input features into the SVM classifier. High levels of discriminability between MLD and TD children after tutoring would suggest that even after performance normalization (Fig. 1c), children with MLD still recruit these brain systems (Fig. 2a) differently from their TD peers, in line with the persistent neural aberration hypothesis. After 8 weeks of tutoring, classification accuracy values discriminating between MLD and TD children based on task-related brain activity patterns dropped to 43.33% and were not significantly different from chance (*P*=0.42) (Fig. 4b).

Altogether, these results provide novel and strong converging evidence for normalization of brain activity patterns after tutoring in children with MLD.

### Functional brain plasticity predicts MLD performance gains

Finally, to assess individual differences of tutoring-induced functional brain plasticity in children with MLD, we computed a brain-based distance metric defined as the Brain Plasticity Index—BPI—(Fig. 4c). Specifically, BPI was calculated for each MLD child by computing a multivariate spatial correlation between pre- and post-tutoring patterns of brain activity, and subtracting it from 1 (Methods). Thus, high levels of BPI reflect greater tutoring-induced functional brain plasticity associated with tutoring. We then examined whether BPI was related to individual differences in performance improvement in children with MLD. Our analysis revealed a significant positive correlation (*r*=0.526, *P*<0.05), such that children with MLD who showed greater tutoring-induced functional brain plasticity, as indexed by BPI, exhibited larger performance gains with tutoring (Fig. 4d).

### Behavioural measures do not predict MLD performance gains

None of the standardized measures (IQ, working memory or math) collected before tutoring were related to performance improvement in children with MLD (all *P*>0.15) (Supplementary Fig. 8). Our results confirm that in contrast to these cognitive assessments^47^, multivariate quantitative measures of functional change in brain activity (Fig. 4c), can provide a more sensitive metric for how well children with MLD respond to an intervention.

## Discussion

Consistent with previous school-based studies, we found that 8 weeks of 1:1 math tutoring focused on strengthening conceptual and procedural knowledge can effectively improve arithmetic problem solving skills in primary-school children with MLD (Fig. 1c, Supplementary Fig. 1). Importantly, we demonstrate that, in parallel with performance normalization^40^, 1:1 tutoring elicits extensive functional brain changes in children with MLD, normalizing their brain activity to the level of neurotypical peers. Prominent differences in brain activation between MLD and TD groups in prefrontal, parietal, ventral temporal–occipital cortices that were evident before tutoring, were entirely absent after tutoring (Fig. 2). Remarkably, tutoring resulted in significant reductions of widespread overactivation in multiple neurocognitive systems important for numerical problem solving (Fig. 3)^6^,^22^,^23^,^24^,^42^, and machine-learning algorithms revealed that brain activity patterns in MLD learners were no longer discriminable from those of their peers after tutoring (Fig. 4b). Finally, children with MLD who displayed greater tutoring-induced functional brain plasticity also exhibited larger performance gains (Fig. 4d), highlighting, for the first time, the behavioural significance of widespread brain changes in response to intervention.

Although MLD was initially conceptualized as a disorder of a single brain region characterized by a localized deficit in the intraparietal sulcus^33^,^48^,^49^, more recently, prominent neurocognitive models of MLD have posited that the disorder stems from more extensive functional aberrations in a distributed network of brain areas encompassing not only posterior parietal, but also prefrontal, as well as ventral temporal–occipital cortices that are known to serve multiple cognitive functions necessary for successful numerical problem solving^1^,^22^. Consistent with this view, before tutoring, we found that, during arithmetic problem solving, children with MLD showed differential and widespread overactivation in multiple neurocognitive systems (Figs 2a and 3a), likely reflecting the need for greater neural resources during arithmetic problem solving^22^,^25^,^27^,^28^,^29^, rather than an inability to activate task-relevant brain areas^32^,^33^,^50^,^51^.

Furthermore, effective intervention in MLD markedly altered aberrant neural responses by inducing functional changes in multiple brain systems (Fig. 2). These functional changes were characterized by reduction of activation following tutoring (Fig. 3b) and were much more widespread than frontoparietal cortex reductions of activation previously reported in brief (typically 5 days) arithmetic training studies in neurotypical adults^52^,^53^, neurodevelopmental studies of neurotypical children and adults^54^, and computer-based training studies in children with low math abilities^55^. This suggests that a comprehensive tutoring program, based on effective school interventions^17^,^19^,^40^ and designed to strengthen both number knowledge and arithmetic fluency^17^,^18^,^19^,^39^,^40^,^41^, can induce changes across distributed brain systems that encompass multiple stages of the information processing hierarchy necessary for successful numerical problem solving^6^,^22^,^42^.

Crucially, our finding of no differences in brain activation between MLD and TD children after tutoring (Fig. 2b) was confirmed by more stringent Bayesian estimation procedures (Supplementary Figs 4 and 5) and, consistent with the neural normalization hypothesis demonstrates that this type of 1:1 tutoring can restore brain activity in children with MLD to the level of their TD peers. This is in stark contrast to the neural compensation hypothesis, which predicts that effective tutoring would be supported by compensatory mechanisms in other brain regions, and we found no evidence of greater activity in any brain area, after tutoring, in children with MLD, compared with their TD peers (Fig. 2b). Furthermore, univariate analysis found no evidence for the persistent neural aberration hypothesis either, as tutoring altered activity levels in all these systems (Fig. 3b), and the brain activity levels were no longer different in the two groups post-tutoring (Fig. 2b).

Computationally rigorous multivariate analyses further substantiated these findings (Fig. 4a,b). First, we tested whether, before tutoring, children with MLD could be discriminated from TD children on the basis of their mean-corrected brain activity patterns. We found that an SVM-based classifier distinguished brain activation patterns in MLD and TD children with an accuracy of 83%; thus, pre-tutoring brain activity patterns during arithmetic problem solving were significantly different in children with MLD from their TD peers. Next, we investigated whether children with MLD could be discriminated from their TD peers based on their brain activity patterns after tutoring. In this case, the classifier accuracy was 43%, indicating that post-tutoring brain activity patterns during arithmetic problem solving were indistinguishable between the two groups. These results demonstrate that MLD and TD groups can be discriminated with high levels of accuracy prior, but not after tutoring, consistent with the neural normalization hypothesis. Critically, they are inconsistent with the persistent neural aberration hypothesis, which would have resulted in high discriminability between brain activity patterns in the MLD and TD groups at both the pre- and post-tutoring sessions. It is important to note that because the brain activity maps used as inputs to the classifier were normalized before classification analysis, discriminability between groups is independent of differences in task-related brain activity levels. These results further highlight the robustness of our findings and suggest that 8 weeks of tutoring not only normalizes brain activation levels, but also activity patterns in children with MLD. Strong multivariate evidence for the neural normalization hypothesis (Fig. 4b) highlights the possibility that this type of 1:1 tutoring program might prove beneficial for future learning in these children, without placing additional burden on effortful processing resources. More generally, the multivariate and quantitatively rigorous approaches used here to investigate learning-induced functional brain plasticity associated with tutoring offer powerful new metrics for detecting individual differences in brain activity patterns associated with interventions.

Finally, leveraging our quantitative approach, and after characterizing the nature of tutoring-induced functional brain plasticity in MLD (Figs 2 and 3), we extended our brain-based multivariate framework to assess individual differences in response to intervention in children with MLD, and assess whether tutoring-induced functional changes are behaviorally meaningful. Specifically, we computed a BPI for each MLD child based on the multivariate spatial correlation between pre- and post-tutoring patterns of brain activity. Remarkably, individual differences in performance gains in children with MLD were strongly correlated with their BPI measures: children who demonstrated greater tutoring-induced functional brain plasticity (that is, greater brain normalization) also exhibited larger performance gains with tutoring (Fig. 4d). In contrast, performance gains in children with MLD were not related to general cognitive abilities (for example, IQ; Supplementary Fig. 8). Furthermore, neuropsychological measures of math abilities before tutoring also did not predict performance gains (Supplementary Fig. 8), further highlighting the robustness and specificity of our brain-behavioural findings.

Our quantitative approach for measuring brain changes following intervention represents a significant advance over previous approaches that have primarily used neuroimaging data to investigate the anatomical loci and direction of change in activation. To our knowledge, this is the first use of a rigorous quantitative approach to assess distributed changes in brain activity pre- and post-intervention in children with MLD, which in turn is essential for more fully characterizing brain mechanisms underlying individual differences in response to intervention.

Further studies are needed to examine transfer to untrained and more complex math problems, and to assess the long-term stability of tutoring-induced functional brain plasticity in individuals with MLD, to make further assertions about the sustained effects of this intervention in the remediation of MLD. Future work should also contrast different types of tutoring programs and intervention groups, to further disentangle cognitive, motivational and psychological factors associated with remediation of learning disabilities. Finally, long-term follow-up studies should also clarify the extent to which the BPI metric developed here can serve as a predictive biomarker of long-term response to treatment^56^.

In conclusion, our study provides the first evidence that 8 weeks of 1:1 targeted cognitive tutoring can successfully remediate both poor math performance as well as aberrant brain responses in primary-school children with MLD. Children with MLD showed strong tutoring-induced functional neuroplasticity across distributed brain systems that support quantity, visuospatial, attention, working memory and cognitive control processes necessary for successful numerical problem solving. Critically, our findings demonstrate for the first time that quantitative measures of functional brain plasticity can significantly predict individual differences in response to treatment in children with MLD, further highlighting the unique potential of systems neuroscience-based approaches to the advancement of educational practice in remediating MLD. More generally, the quantitative framework developed here is likely to be useful for investigating brain plasticity associated with response to intervention in other forms of learning disabilities as well as in psychiatric, neurological, neurodevelopmental and neurodegenerative disorders^56^.

## Methods

### Participants

A total of 46 children in their third grade of schooling (ages 7.5–9.6 years) were recruited from multiple school districts in the San Francisco Bay Area. All participants were right-handed and without medical, neurological or psychiatric illness. Informed written consent was obtained from the legal guardian of the child and all study protocols were approved by the Stanford University Review Board. All participants were volunteers and were treated in accordance with the American Psychological Association ‘Ethical Principles of Psychologists and Code of Conduct'.

A total of 16 children were excluded from the study because they did not meet inclusion criteria for (i) in-scanner motion parameters (total frames interpolated <20%) and adequate whole-brain coverage, (ii) in-scanner accuracy performance (>50%) and (iii) neuropsychological scores (Supplementary Methods). Thus, the analyses presented in this study used a sample of thirty children, 15 of whom were characterized as having MLD, and 15 who were determined to be TD control children. This sample size was based on previously published studies of children with MLD^27^,^29^,^30^,^33^. Bayesian analyses confirmed that before tutoring, between-group differences in brain activation could be detected with a confidence interval of 95% using this sample size (Supplementary Figs 4 and 5).Additional details regarding participant selection, demographic as well as cognitive assessments, are presented in the Supplementary Methods.

### Overall study design

Figure 1a illustrates the tutoring study design. Demographic, neuropsychological, cognitive and brain-imaging measures were acquired from each participant prior to tutoring. Before commencing 8 weeks of 1:1 math tutoring, participants underwent an MRI scan session, which included both a fMRI as well as a structural MRI acquisition protocol. The fMRI tasks consisted of an arithmetic verification task (Addition task), where the child had to verify the validity of addition equations (for example, 3+4=7), and a number identity verification task (Control task), where the child had to assess the validity of number identity expressions (for example, 7=7). After successful completion of the MRI scan session, children started the 8-week math tutoring program. Tutoring sessions occurred three times per week and were each approximately 40–50 min in duration. A second MRI scan session using the same protocols took place after the 8 weeks of tutoring.

### Neuropsychological assessments

Each child participated in a neuropsychological assessment session in which they were tested on the Wechsler Abbreviated Scale of Intelligence (WASI)^57^, the Wechsler Individual Achievement Test (WIAT-II)^58^ and the Working Memory Test Battery for Children (WMTB-C)^59^. Full-scale IQ was determined using the WASI; academic achievement in reading and mathematics was assessed using the WIAT-II; working memory was assessed using the WMTB-C. These standardized measures were acquired before tutoring and were not readministered because of their limited validity when repeated within a year. The Scale for Early Mathematics Anxiety was also administered before and after tutoring in both groups^60^,^61^. Parametric tests were used for all analyses (two-sided *t*-tests), as data were normally distributed in both samples (all *K*–*S* tests for normality *P* values were >0.155 for the MLD group and *P*>0.625 for the TD group).

### Tutoring sessions

All children took part in an 8-week math tutoring program adapted from MathWise^17^,^19^,^40^. The tutoring combined conceptual instruction with speeded retrieval of arithmetical facts. The focus of the tutoring included strengthening of number knowledge (for example, cardinality) and relations within and between operations (for example, commutativity and inverse relation between addition and subtraction) that facilitate the use of sophisticated counting procedures and retrieval-based processes. The tutoring also incorporated a strategic practice component, that is important for building automaticity^40^, and decreasing load on cognitive resources (for example, working memory and non-verbal reasoning)^62^. This practice was designed to promote quick responding and use of efficient counting procedures to generate as many correct responses as possible, which in turn supports the formation and strengthening of representations in long-term memory^63^,^64^. Similarly to MathWise^40^, the tutoring involved a total of 15–20 h of tutoring. Differently from MathWise, for which tutoring occurred over the course of 15–16 weeks, the present tutoring was condensed to 8–9 weeks. Thus, the current tutoring had longer sessions—from 40 min to 50 min—to equate overall time on tutoring. Specifically, the present tutoring consisted of 22 lessons of increasing difficulty (details of each lesson and the tutoring material are described in the Supplementary Methods).

### Outside-the-scanner arithmetic production task

On the day of the MRI session, before entering the scanner, children also performed an arithmetic production task (for example, 4+3=?). A total of 24 problems, involving random pairs of integers from 2 to 19, with sums ranging from 6 to 25 were presented to the child on a computer screen. The larger operand was equally likely to appear in the first or second position, as in the arithmetic verification task performed inside the scanner. The child was required to accurately solve each problem without the use of paper and pencil and to verbally state the answer out loud. For each problem, the experimenter recorded the child's response verbatim on paper. Proportion of correctly solved problems was computed as the outcome measure of interest. Three children (one in the MLD group, and two in the TD group) did not complete this task due to time constraints. Parametric tests were used for this analysis (two-sided *t*-tests), as data were normally distributed in both samples (all *K*–*S* tests for normality *P* values were >0.628 for the MLD group and >0.320 for the TD group for both pre- and post-tutoring session).

### Brain imaging

*Functional MRI data acquisition*. fMRI data were acquired using whole-brain imaging with a T2*-sensitive gradient echo spiral in/out pulse sequence at a Signa LX (GE Medical Systems) 3T scanner with the following parameters: echo time (*TE*)=30 ms, repetition time (*TR*)=2 s, flip angle=80°, field-of-view=200 mm, 29 axial-oblique slices parallel to the AC–PC, dimensions 3.125 × 3.125 × 4 mm with 0.5-mm skip. To reduce blurring and signal loss from field inhomogeneity, an automated high order shimming method based on a spiral acquisition was used prior to the acquisition of functional MRI scans^65^. Cushions were placed around participants' heads to minimize head movement.

*Functional MRI tasks*. The arithmetic verification task—Addition task—took place inside the scanner during task-based fMRI acquisition. This task consisted of two runs of arithmetic problem solving during which the child had to verify addition equations (for example, 3+4=7). Problems were presented in a fast event-related fMRI design with 12 single-digit problems per run. In each run, problems were presented horizontally in green lettering on a black background. In half of the problems, the answers presented were correct (for example, 2+4=6); in the remaining half, the answers presented deviated from the correct solution by ±1 or ±2 (for example, 3+5=7). Arithmetic problems with 1 or 0 as operands were excluded. The larger operand was equally likely to appear in the first or second position. Each trial started with a fixation asterisk that lasted for 0.5 s. Then, the problem was presented for a maximum of 9.5 s, during which time the child could make the response. The participant used a response box to indicate if the answer was correct or not. After the response, the problem disappeared from the screen and a black screen appeared until the time window was filled to 9.5 s. A set of 12 non-arithmetic problems was also presented during each run and they constituted the Control task. These problems consisted of number identity verifications (for example, 7=7) and were randomly interspersed with the arithmetic trials. Invalid trials were counterbalanced as in the arithmetic verification task (that is, answers deviated from the correct solution by ±1 or ±2). This condition served as the control task for fMRI data analyses to better isolate brain activity solely related to arithmetic problem solving, rather than sensory or number processing, decision making and response selection. The task design also included a total of six rest periods—10 s each —, which occurred at jittered intervals during each run to achieve an optimal event-related fMRI design^66^. The rest periods were not explicitly modelled. Accuracy and mean of median reaction times of correctly solved problems were computed separately for each participant for each task-based condition (that is, arithmetic verification and number identity verification). Parametric tests were used for these analyses (two-sided *t*-tests), as data were normally distributed in both samples (all *K*–*S* tests for normality *P* values were >0.620 for the MLD group and >0.657 for the TD group).

### Structural MRI data acquisition

High-resolution T1-weighted images were acquired in each child at both scan sessions (that is, pre- and post-tutoring), to facilitate anatomical co-registration of fMRI maps. A spoiled-gradient-recalled inversion recovery three-dimensional (3D) MRI sequence with the following parameters was used: *I*=300 ms, *TR*=8.4 ms; *TE*=1.8 ms; flip angle=15°; 22-cm field of view; 132 slices in coronal plane; 256 × 192 matrix; 2 NEX, acquired resolution=1.5 × 0.9 × 1.1 mm.

### Functional MRI data analysis

*Functional MRI preprocessing*. Data were analysed using SPM8 (http://www.fil.ion.ucl.ac.uk/spm/). The first five volumes were not analysed to allow for signal equilibration. A linear shim correction was applied separately for each slice during reconstruction using a magnetic field map acquired automatically by the pulse sequence at the beginning of the scan^67^. Images were realigned to correct for motion, corrected for errors in slice-timing, co-registered to each individual's structural T1 images, spatially transformed to standard stereotaxic space (based on the Montreal Neurologic Institute coordinate system), resampled every 2 mm using sinc interpolation, and smoothed with a 6 mm full-width half-maximum Gaussian kernel to decrease spatial noise prior to statistical analysis. For co-registration, the individual's highest quality-rated (that is, either pre- or post-tutoring) structural MRI sequence was used.

Translational movement in millimeters (*x*,*y*,*z*), and rotational motion in degrees (pitch, roll, yaw) were calculated based on the SPM8 parameters for motion correction of the functional images of each subject. Mean scan-to-scan displacement of movement did not exceed 1 mm for all participants in either session (that is, pre- or post-tutoring). To correct for deviant volumes resulting from spikes in movement, we used de-spiking procedures similar to those implemented in AFNI^68^. Volumes with movement exceeding 0.5 voxels (1.562 mm) or spikes in global signal exceeding 5% were interpolated using adjacent scans. No >12% of total volumes per run were repaired in either group. Critically, no differences between the groups on the total percent of volumes repaired were evident either pre- or post-tutoring: pre-tutoring (*P*=0.52), post-tutoring (*P*=0.09), or between sessions in either group: MLD (*P*=0.62), TD (*P*=0.8). Translational movement parameters (*x*,*y*,*z*), and rotational movement parameters (roll, pitch and yaw) did not differ between the groups at either pre- or post-tutoring sessions (all *P*>0.06). Within each group, movement parameters did not differ between the pre- and post-tutoring sessions (all *P*>0.09). Finally, mean scan-to-scan displacement also did not differ between the groups, or sessions (all *P*>0.12).

### Univariate analyses

*General linear model*. Task-related brain activation was identified using the GLM implemented in SPM8. At an individual level, brain activity related to task conditions was modeled using a boxcar function of 9.5 s (that is, the whole length of any given trial) with a canonical hemodynamic response function and a temporal derivative to account for voxel-wise latency differences in hemodynamic response. Voxel-wise contrast and *t*-statistic images were generated for each participant by contrasting arithmetic verification (Addition) versus number identity verification (Control) conditions and were averaged across the two runs. Both correct and incorrect trials were modeled in the GLM (four sub-conditions: Addition correct, Control correct, Addition incorrect, Control incorrect). The final voxel-wise contrast and *t*-statistic maps were generated on the first two sub-conditions only: Addition correct and Control correct.

At a group level, differences in brain activation between the MLD and TD groups were compared at both sessions (pre- and post-tutoring separately) using a *t*-test on contrast images of the Addition versus Control conditions. Differences in brain activation between pre- and post- tutoring sessions were compared within each group using a *t*-test on the same contrast images. In both analyses effects were measured at the whole-brain level and significant clusters of activation were identified using a height threshold of *P*<0.01, with family-wise error (FWE) correction for multiple spatial comparisons at the cluster level (*P*<0.01, spatial extent 128 voxels) based on Monte Carlo simulations.

### Multivariate analyses

*Multivariate classification analysis*. We used multivariate classification analysis to assess whether functional brain activity patterns could be used to discriminate children with MLD from their TD peers either before or after tutoring (Fig. 4a). Brain activity patterns (*t*-maps) elicited during arithmetic problem solving were used as the input (features) to a pattern-based classifier. The maps were masked with brain areas in which the MLD and TD groups differed in activation at pre-tutoring (Fig. 2a, Supplementary Table 3). These maps were first *z*-transformed (demeaned and scaled by variance), thus ensuring that group differences were independent of differences in task-related activity levels. The *z*-transformed activity maps were used as input features to a pattern-based SVM classifier^46^ and LOOCV procedures were used to assess discriminability of task-related activation patterns between the MLD and TD groups. The classifier distinguishes MLD from TD children by making a classification decision based on values of the linear combination of these features. We employed a widely used linear-classifier (SVM Classification)^46^ that was best suited for our purpose of classification-based models that are based on large number of features (brain based), but a small number of training samples (*n*=30 in our case), which is typically the case of human brain neuroimaging studies. We used the Matlab package libSVM (http://www.csie.ntu.edu.tw/~cjlin/libsvm) to fit the classifier. LOOCV was used to measure the performance of the classifier in distinguishing MLD from TD children, based on patterns of brain activity. In LOOCV, data are divided into *N* folds (here, *n*=30). A classifier is built using *n*−1 folds, leaving out one sample. The left out sample is then classified using this classifier, and the Classification Accuracy (CA) is noted. The above procedure is repeated *N* times by leaving out one sample each time, and finally an average CA is computed. Permutation tests (10,000 permutations of class labels) were conducted to arrive at *P* values associated with CA.

*Multivariate distance analysis*. We used a multivariate distance analysis to assess the behavioral significance of tutoring-induced functional brain plasticity (Fig. 4c). Specifically, we computed a brain-based distance metric defined as the BPI. BPI was calculated for each individual with MLD by computing a multivariate spatial correlation between pre-tutoring patterns of brain activity and post-tutoring patterns of brain activity. As in the previous section, these maps were masked with brain areas in which the MLD and TD groups differed in activation at pre-tutoring (Fig. 2a, Supplementary Table 3), and were *z*-transformed (demeaned and scaled by variance), thus ensuring that effects were independent of differences in task-related activity levels. These values were then subtracted from 1, to facilitate easier conceptualization of the measure: the more plastic the brain, the higher the BPI. Parametric correlation analyses were then performed between the computed BPI values and performance gains (that is, calculated by subtracting pre-tutoring accuracy values from post-tutoring accuracy values) to investigate whether BPI was related to individual differences in performance improvement in children with MLD.

## Additional information

**How to cite this article:** Iuculano, T. *et al*. Cognitive tutoring induces widespread neuroplasticity and remediates brain function in children with mathematical learning disabilities. *Nat. Commun*. 6:8453 doi: 10.1038/ncomms9453 (2015).

## Supplementary Material

Supplementary Information Supplementary Figures 1-8, Supplementary Tables 1-7, Supplementary Methods, and Supplementary References
