# Supplementary material for: Cognitive tutoring induces widespread neuroplasticity and remediates brain function in children with mathematical learning disabilities
Source: Nat Commun. 2015 Sep 30;6:8453. doi: 10.1038/ncomms9453 (PMC4598717; doi:10.1038/ncomms9453)
Supplement: Supplementary Information — Supplementary Figures 1-8, Supplementary Tables 1-7, Supplementary Methods, and Supplementary References [file ncomms9453-s1.pdf]

Supplementary Figure 1.

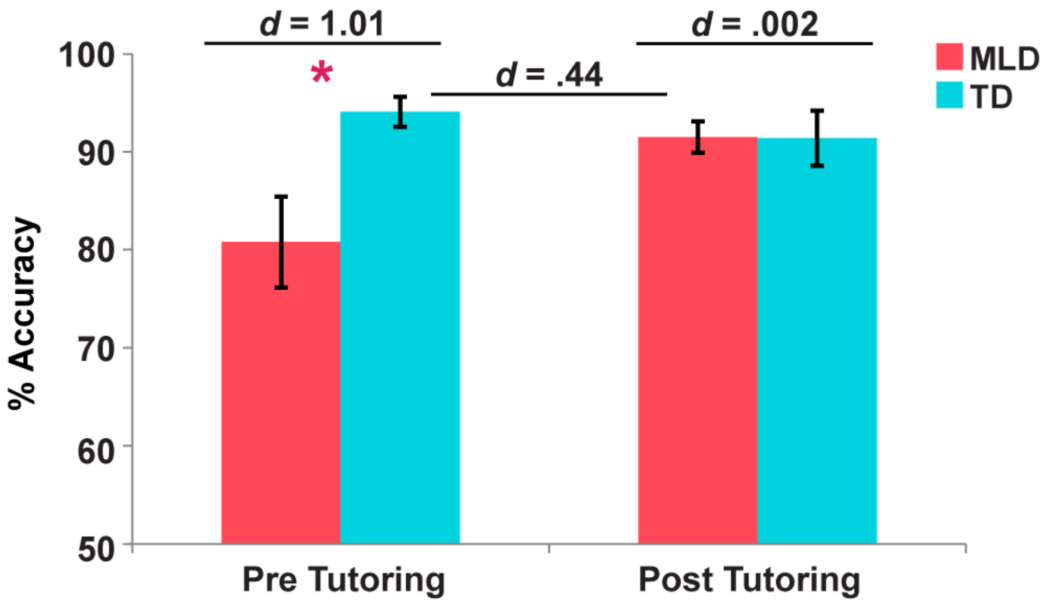

<u>Supplementary Figure 1.</u> Outside-the-scanner arithmetic production task. Performance normalization on the arithmetic production task in children with MLD (*n* = 14) after 8 weeks of math tutoring plotted against TD (*n* = 13) children's performance at pre-tutoring and post-tutoring sessions. Error bars indicate one standard error of the mean (SEM). *$P < .05$, significant by independent samples *t*-test. Effect sizes for group differences are shown as Cohen's *d*.

Supplementary Figure 2.

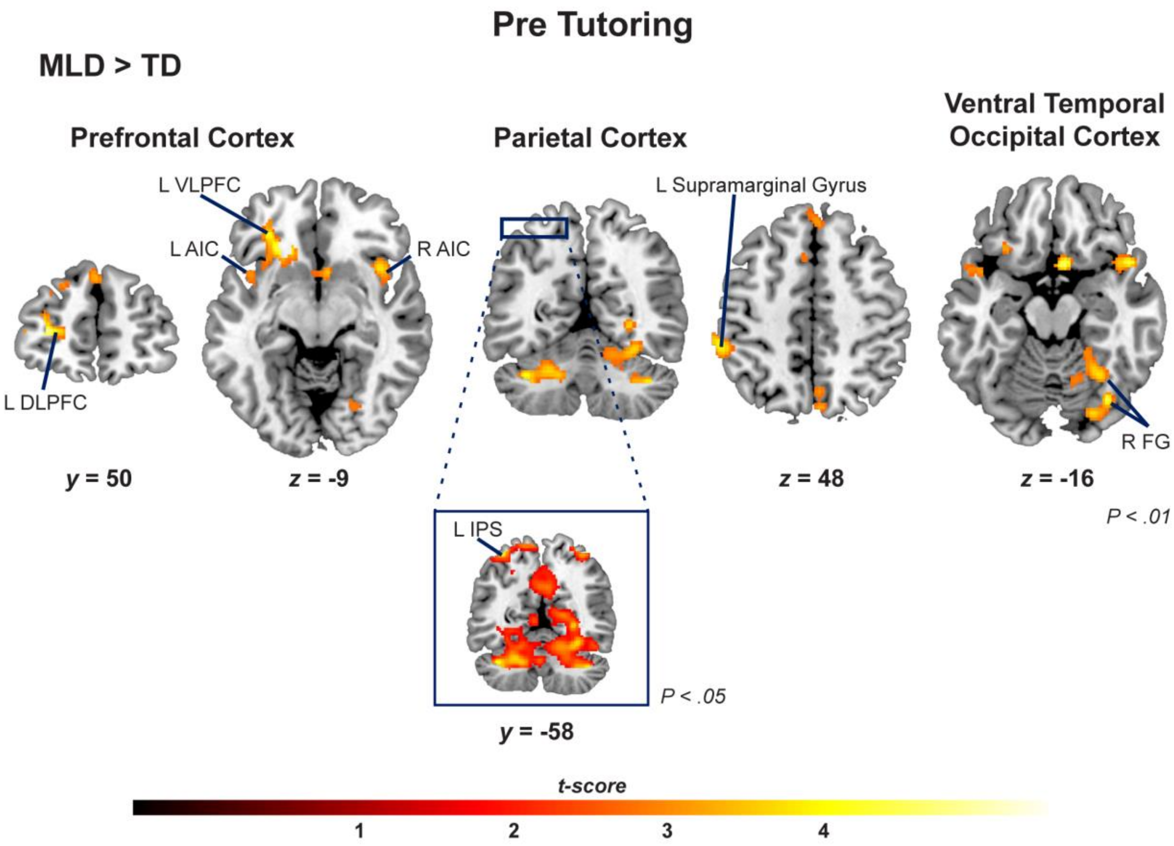

<u>Supplementary Figure 2.</u> **Functional activation differences between MLD and TD children before tutoring – Controlling for accuracy scores at pre-tutoring.** Prior to tutoring, compared to TD children (n = 15), children with MLD (n = 15) showed significant differences in brain activation levels in multiple areas in the Prefrontal Cortex, including the left Dorsolateral and Ventrolateral Prefrontal Cortices (DLPFC and VLPFC), and the bilateral Anterior Insular Cortices (AIC); in the Parietal Cortex encompassing the left Supramarginal Gyrus, but not the left Intraparietal Sulcus (IPS), which only showed a group-effect at a lower threshold (height threshold $P < .05$, extent threshold $P < .01$, significant by whole brain voxel-wise independent samples t-test); and in the Ventral Temporal-Occipital Cortex including the right Fusiform Gyrus (FG). Height threshold $P < .01$, extent threshold $P < .01$, significant by whole brain voxel-wise independent samples t-test.

Supplementary Figure 3.

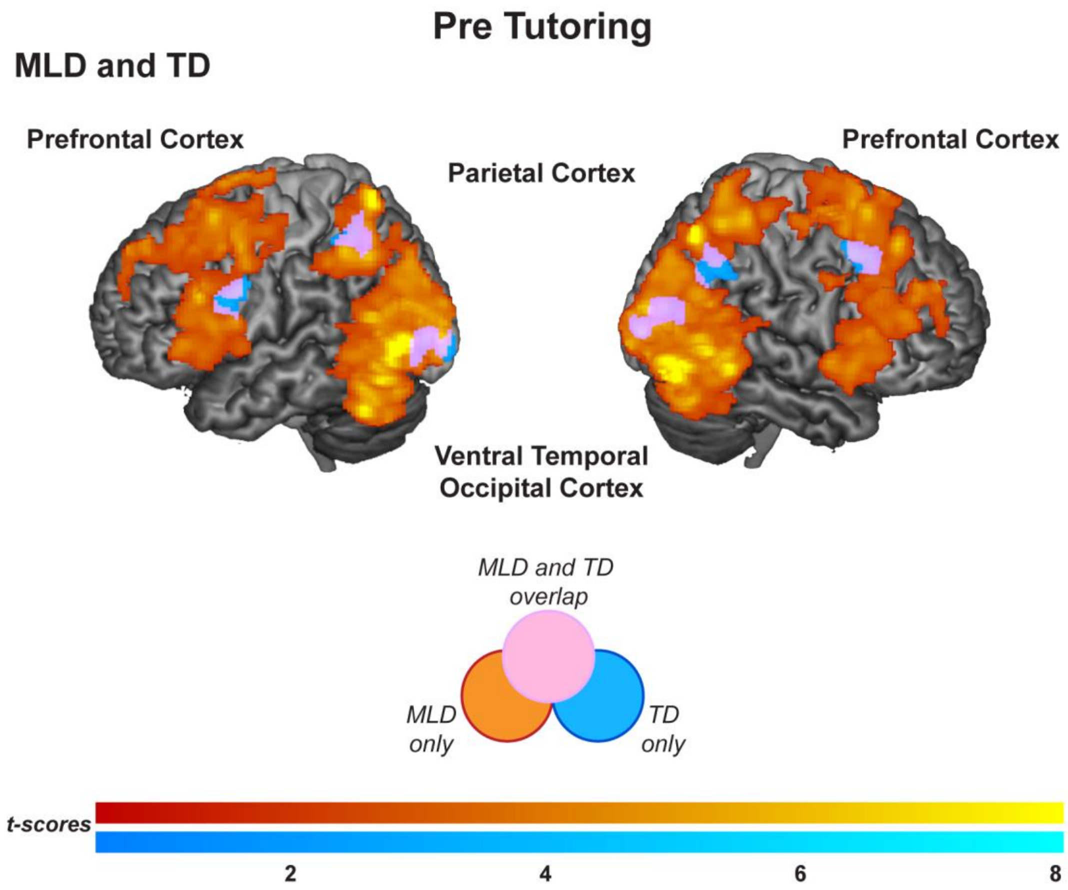

<u>**Supplementary Figure 3.**</u> **Overlap in activation in the MLD and TD groups prior to tutoring.** Children with MLD (n = 15) showed widespread activation in multiple cortical areas encompassing the prefrontal, parietal, and ventral temporal-occipital cortices. TD children (n = 15) activated these cortical areas as well, but responses were more focal. Brain areas that showed activation only in the MLD group are shown in orange; TD only activation in blue; while overlap in MLD and TD activation is shown in pink. Height threshold $P < .01$, extent threshold $P < .01$ significant by whole brain voxel-wise one-sample t-tests.

Supplementary Figure 4.

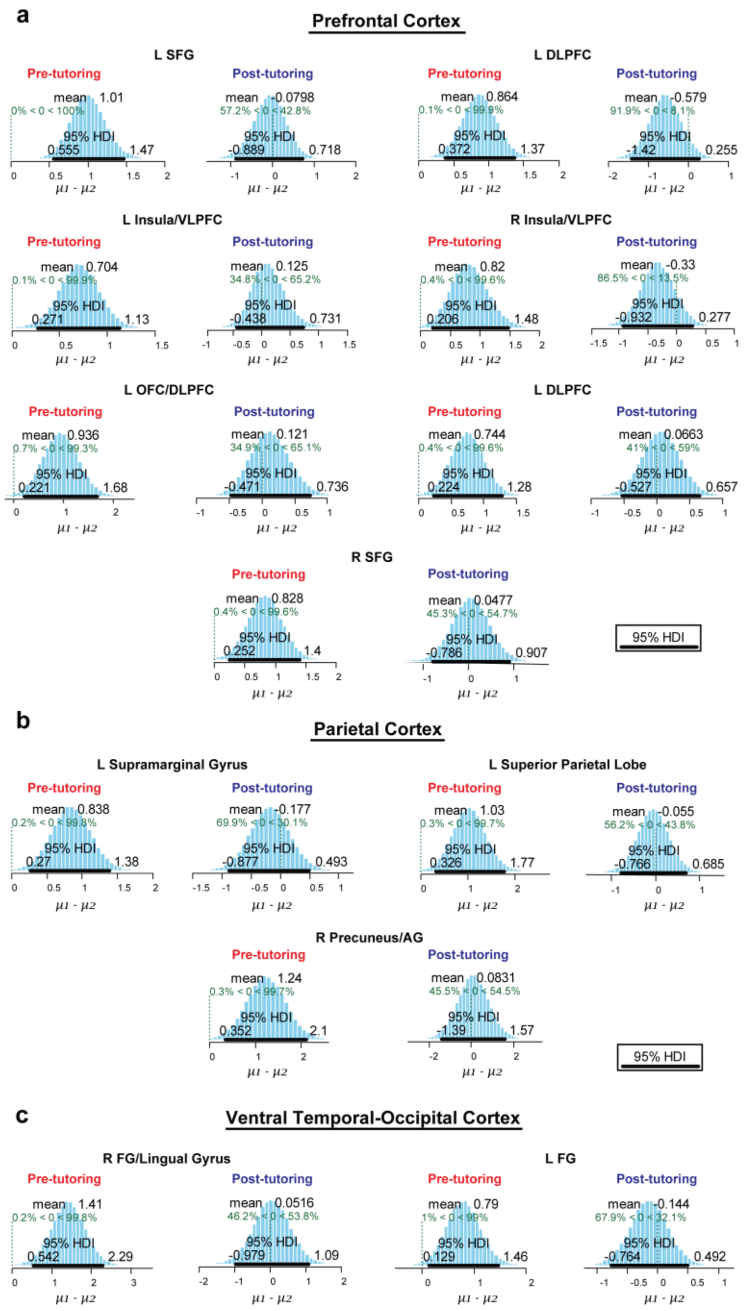

<u>Supplementary Figure 4.</u> Bayesian analysis of group differences at pre-tutoring and post-tutoring sessions – Cortical areas. Pre-tutoring session: 95% confidence intervals of differences ($\mu 1 - \mu 2$) in mean *beta* parameter estimates between MLD (n = 15) and TD (n = 15) groups are centered on 1 (shown in red). **Post-tutoring session:** 95% confidence intervals of differences in means ($\mu 1 - \mu 2$) are centered on 0 (shown in blue). *Abbreviations: SFG = Superior Frontal Gyrus; DLPFC = Dorsolateral Prefrontal Cortex; VLPFC = Ventrolateral Prefrontal Cortex; OFC = Orbitofrontal Cortex; AG = Angular Gyrus; HDI = High Dimensional Inference.*

Supplementary Figure 5.

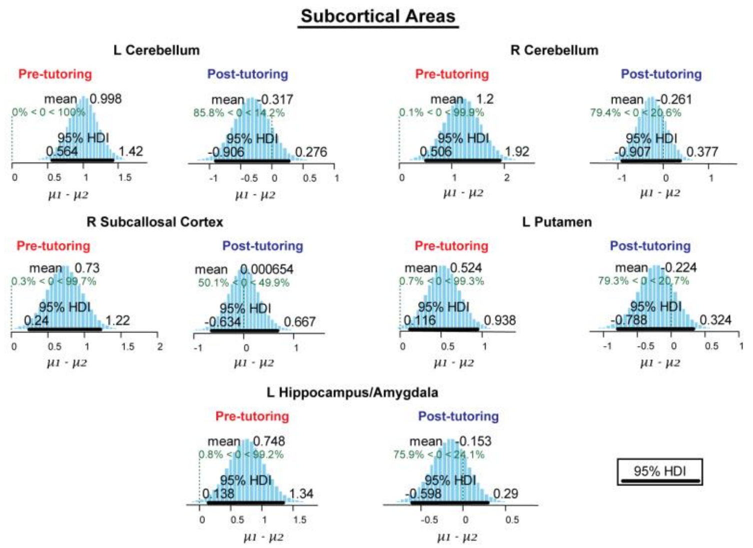

<u>**Supplementary Figure 5.**</u> **Bayesian analysis of group differences at pre-tutoring and post-tutoring sessions – Subcortical areas. Pre-tutoring session:** 95% confidence intervals of differences ($\mu 1 - \mu 2$) in mean *beta* parameter estimates between MLD (n = 15) and TD (n = 15) groups are centered on 1 (shown in red). **Post-tutoring session:** 95% confidence intervals of differences in means ($\mu 1 - \mu 2$) are centered on 0 (shown in blue). *Abbreviations: HDI = High Dimensional Inference.*

Supplementary Figure 6.

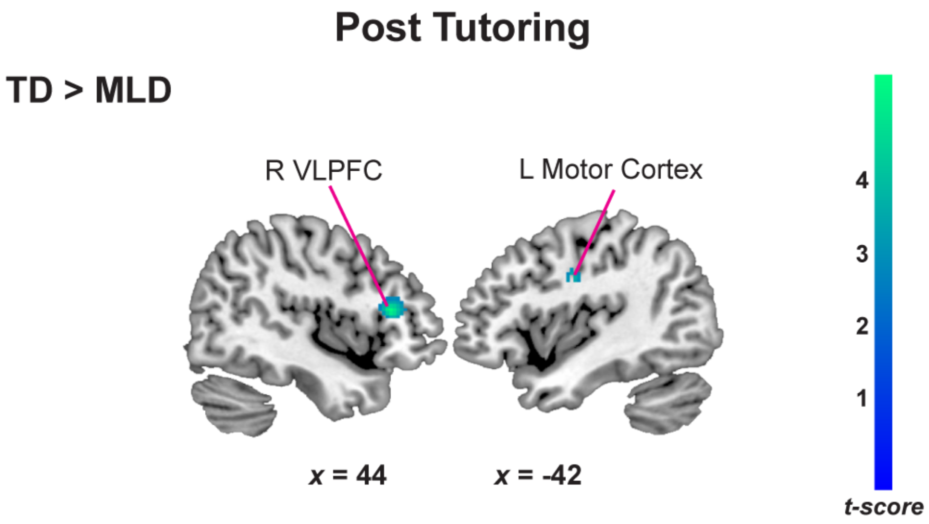

<u>Supplementary Figure 6.</u> Post-tutoring differences in activation between TD and MLD groups. Post-tutoring, the only difference in brain activation between the two groups was that, compared to MLD children (n = 15), TD children (n = 15) showed greater brain activity in the right Ventrolateral Prefrontal Cortex (VLPFC) and in the left Motor Cortex. No brain regions showed greater activation in children with MLD, compared to TD children. Height threshold $P<$ .01, extent threshold $P < .01$, significant by whole brain voxel-wise independent samples t-test.

Supplementary Figure 7.

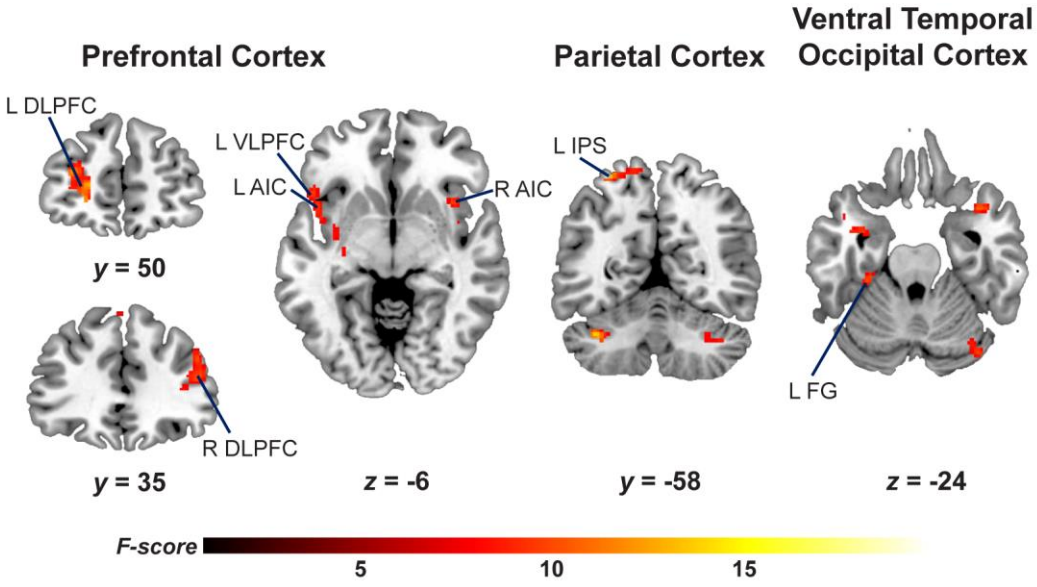

<u>**Supplementary Figure 7.**</u> **Group by tutoring-session interaction.** Activation differences between MLD (*n* = 15) and TD (*n* = 15) children that were modulated by tutoring-session (pre, post) were evident in the Prefrontal Cortex, including the bilateral Dorsolateral Prefrontal Cortices (DLPFC), the left Ventrolateral Prefrontal Cortex (VLPFC), and the bilateral Anterior Insular Cortices (AIC); in the Parietal Cortex, encompassing the left Intraparietal Sulcus (IPS); and in the Ventral Temporal-Occipital Cortex including the left Fusiform Gyrus (FG). Height threshold $P < .01$, extent threshold $P < .01$, significant by whole brain voxel-wise 2x2 mixed design analysis of variance (ANOVA).

Supplementary Figure 8.

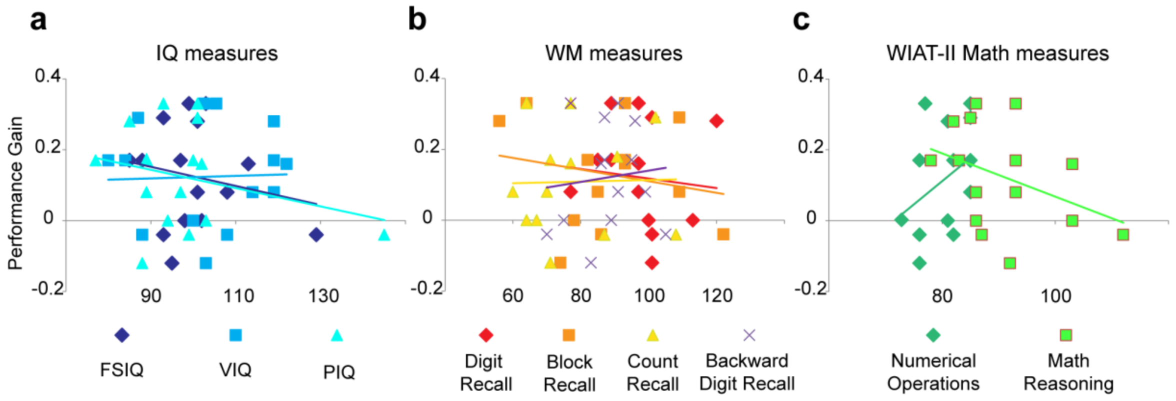

<u>**Supplementary Figure 8.**</u> **Relation between neuropsychological measures and tutoring-related performance gains in children with MLD.** None of the standardized domain-general cognitive measures collected at pre-test was related to performance improvement in children with MLD (n = 15). (**a**) IQ measures. *Full-scale IQ (FSIQ)*: r = .199, P = .48; *Verbal IQ (VIQ)*: r = -.034, P = .9; *Performance IQ (PIQ)*: r =.269, P = .33; (**b**) Working Memory (WM) measures. *Digit Recall*: r = .1, P = .74; *Block Recall*: r = .2, P = .51; *Count Recall*: r = -.23, P = .94; *Backward Digit Recall*: r = -.1, P = .73. (**c**) WIAT-II Math measures. *Numerical Operations*: r = -.387, P = .16, and *Math Reasoning*: r = .389, P = .15.

Supplementary Table 1.

Measure	MLD (N = 15)	TD (N = 15)	*P*-value
**Male to Female ratio**	6:9	7:8	
**Age (years)**	8.65 (±0.47)	8.54 (±0.49)	0.52
**WASI scale**			
Verbal IQ	103.40 (±13.80)	106.60 (±15.68)	0.56
Performance IQ	97.53 (±15.08)	106.73 (±11.18)	0.07
Full-Scale IQ	100.33 (±10.67)	107.47 (±11.05)	0.08
**WIAT-II scale**			
Numerical Operations	80.93 (±4.25)	105.80 (±9.53)	0.00001***
Math Reasoning	90.80 (±9.19)	106.20 (±9.36)	0.00001***
Word reading	100.20 (±9.67)	106.27 (±9.01)	0.09
Reading Comprehension	102.27 (±7.87)	108.07 (±11.11)	0.11
**WMTB - C**			
Digit Recall	97.71 (±10.82)	99.13 (±17.75)	0.79
Block Recall	88.00 (±18.39)	93.20 (±17.59)	0.31
Count Recall	77.61 (±15.07)	86.71 (±14.61)	0.15
Backwards Digit Recall	87.93 (± 9.55)	92.93 (±15.21)	0.23

WASI = Wechsler Abbreviated Scale of Intelligence; WIAT –II = Wechsler Individual Achievement Test – Second Edition; WMTB – C = Working Memory Test Battery for Children; *df* = (1,28) for all statistics.

<u>**Supplementary Table 1.**</u> **Demographic, IQ, and diagnostic measures in MLD and TD groups.** The groups were matched on age, IQ, reading and working memory abilities. The two groups differed significantly on both math achievement measures of the WIAT-II scale (i.e. Numerical Operations and Math Reasoning) (both $P < .00001$).

Supplementary Table 2.

MLD	***t*-score**	***P*-value**
Numerical Operations - FSIQ	-7.467	0.00001***
Math Reasoning – FSIQ	-5.079	0.00001***
Word Reading – FSIQ	-0.039	0.969
Reading Comprehension - FSIQ	0.832	0.419
TD		
Numerical Operations - FSIQ	-0.449	0.661
Math Reasoning – FSIQ	-0.385	0.706
Word Reading – FSIQ	-0.304	0.765
Reading Comprehension - FSIQ	0.151	0.882

df (1,14) for all statistics.

Supplementary Table 2. Math and reading discrepancy scores in MLD and TD groups. Children with MLD showed a significant discrepancy between WIAT-II math achievement measures and their Full-Scale IQ (FSIQ) scores (both $P < .00001$). This discrepancy was not evident on their WIAT-II reading achievement measures (all $P > .42$), and it was not present in the TD group for either the math or reading measures (all $P > .66$).

Supplementary Table 3.

**Region**	**MNI coordinates** *x*	*y*	*z*	**Cluster size**	**Max *t***	**Cohen's *d***
MLD > TD						
**Prefrontal Cortex**						
L SFG	-14	26	60	808	4.83	1.87
L DLPFC	-30	50	6	190	4.20	1.24
L Insula/VLPFC	-30	30	0	707	4.02	1.02
R Insula/VLPFC	40	12	-12	287	3.72	1.12
R OFC/DLPFC	12	56	6	243	3.53	0.90
L DLPFC	-20	44	36	502	3.47	1.02
R SFG	20	26	58	133	3.32	0.80
**Parietal Cortex**						
L Supramarginal Gyrus	-58	-36	46	131	4.06	1.21
L Superior Parietal Lobe	-34	-58	60	193	3.65	1.12
R Precuneus/AG	6	-76	46	660	3.60	1.02
**Ventral Temporal-Occipital Cortex**						
R Fusiform Gyrus/Lingual Gyrus[†]	24	-80	-14	1382	4.13	0.83
L Fusiform Gyrus	-30	-32	-24	141	3.34	0.95
**Subcortical Areas**						
L Cerebellum	-36	-62	-34	526	4.65	1.66
R Cerebellum	36	-68	-32	[†]	4.52	1.37
R Subcallosal Cortex	6	12	-12	261	4.22	1.19
L Putamen[††]	-30	-16	-8	277	3.37	0.90

L Hippocampus/Amygdala	-28	-20	-20	[††]	3.28	1.01
TD > MLD						
No significant voxels						

Abbreviations: SFG = Superior Frontal Gyrus; DLPFC = Dorsolateral Prefrontal Cortex; VLPFC = Ventrolateral Prefrontal Cortex; OFC = Orbitofrontal Cortex; AG = Angular Gyrus. Note: Height threshold P < .01, corrected for multiple comparisons using Monte Carlo Simulation at P < .01 extent. Effect sizes for group differences are shown as Cohen's d. [†,††]*These are anatomically distinct sub-peaks of the same activated cluster.*

Supplementary Table 3. **Pre-tutoring differences in brain activation between MLD and TD groups.** Brain regions that showed significantly greater activation during arithmetic problem solving in children with MLD, compared to TD children, prior to tutoring. No brain areas showed greater activation in TD children, compared to the MLD group.

Supplementary Table 4.

Region	MNI coordinates *x*	*y*	*z*	Cluster size	Max *t*
MLD					
**Prefrontal Cortex**					
L DLPFC/IFG/VLPFC	-52	20	30	1431	6.57
L SFG	-8	12	58	1209	6.21
R SFG/MFG/DLPFC	32	2	66	249	5.30
R Insula/DLPFC	36	2	-6	1663	5.08
L Insula/OFC/VLPFC	-30	28	0	491	4.74
**Parietal Cortex**					
R Superior Parietal Lobe	36	-64	54	369	11.38
L Superior Parietal Lobe	-26	-50	46	1036	6.49
**Ventral Temporal-Occipital Cortex**					
R Fusiform Gyrus/Lingual Gyrus/LOC[††]	18	-74	-16	11345	13.23
L Visual Cortex/Fusiform Gyrus/LOC	-22	-68	-20	†	7.58
**Subcortical Areas**					
L Cerebellum	-36	-60	-30	†	8.55
R Cerebellum	36	-64	-28	†	6.04
TD					
**Prefrontal Cortex**					
L DLPFC/IFG/VLPFC/Precentral Gyrus	-36	2	28	210	4.42
**Parietal Cortex**					
R Superior Parietal Lobe/hIP3	30	-54	38	158	3.05

**Ventral Temporal-Occipital Cortex**					
L Visual Cortex/Fusiform Gyrus/LOC	-36	-84	-4	286	4.53

Abbreviations: DLPFC = Dorsolateral Prefrontal Cortex; IFG = Inferior Frontal Gyrus; VLPFC = Ventrolateral Prefrontal Cortex; SFG = Superior Frontal Gyrus; MFG = Middle Frontal Gyrus; OFC = Orbitofrontal Cortex; LOC = Lateral Occipital Cortex. Note: Height threshold P < .01, corrected for multiple comparisons using Monte Carlo Simulation at P < .01 extent. [†]*These are anatomically distinct sub-peaks of the same activated cluster.*

Supplementary Table 4. Pre-tutoring brain activation in MLD and TD groups. Brain regions that showed significant activation during arithmetic problem solving before tutoring, in children with MLD and in TD children, respectively.

Supplementary Table 5.

	**MNI coordinates**					
**Region**	***x***	***y***	***z***	**Cluster size**	**Max *t***	**Cohen's *D***
MLD > TD						
No significant voxels						
TD > MLD						
**Prefrontal Cortex**						
R VLPFC	44	28	14	192	4.87	1.25
L Motor Cortex	-42	-8	32	281	3.41	0.99

Abbreviations: VLPFC = Ventrolateral Prefrontal Cortex. Note: Height threshold P < .01, corrected for multiple comparisons using Monte Carlo Simulation at P < .01 extent. Effect sizes for group differences are shown as Cohen's d.

Supplementary Table 5. Post-tutoring differences in brain activation between TD and MLD groups. Brain regions that showed significantly greater activation during arithmetic problem solving in TD children, compared to children with MLD at post-tutoring. No brain areas showed greater activation in MLD children, compared to the TD group.

Supplementary Table 6.

Region	MNI coordinates *x*	*y*	*z*	Cluster size	Max *F*
**Prefrontal Cortex**					
R Insula	40	12	-12	288	17.30
L SFG†	-8	14	58	452	15.73
L IFG/VLPFC††	-54	10	8	1382	13.82
L DLPFC	-20	50	8	209	12.77
R MFG/DLPFC	46	30	30	286	12.64
L Insula	-36	-4	-2	††	10.25
R SFG	2	30	58	†	7.31
**Parietal Cortex**					
L Superior Parietal Lobe	-30	-56	64	200	17.10
**Ventral Temporal Occipital Cortex**					
L Fusiform Gyrus†††	-30	-32	-24	334	9.53
**Subcortical Areas**					
L Cerebellum	-36	-62	-34	234	20.26
R Cerebellum	36	-70	-30	409	20.09
L Hippocampus/Amygdala	-28	-18	-20	†††	12.13
L Putamen	-30	-16	-8	†††	8.34

Abbreviations: SFG = Superior Frontal Gyrus; IFG = Inferior Frontal Gyrus; VLPFC = Ventrolateral Prefrontal Cortex; DLPFC = Dorsolateral Prefrontal Cortex; MFG = Middle Frontal Gyrus. Note: Height threshold P < .01, corrected for multiple comparisons using Monte Carlo Simulation at P < .01 extent. †, ††, †††*These are anatomically distinct sub-peaks of the same activated cluster.*

Supplementary Table 6. Group by Tutoring-session interaction. Brain regions that showed a significant interaction between tutoring-session (pre and post), and group (MLD and TD).

Supplementary Table 7.

Region	MNI coordinates *x*	*y*	*z*	Cluster size	Max *t*	Cohen's *d*
Pre > Post						
**Prefrontal Cortex**						
L DLPFC	-22	52	10	518	8.48	2.21
R DLPFC/Insula/VLPFC†	44	30	32	15111	7.02	1.38
L MFG/Precentral Gyrus	-48	6	44	1659	5.73	1.40
L SFG	-4	12	58	697	4.14	1.04
L OFC/VLPFC/Insula	-50	22	-10	190	4.13	1.06
**Parietal Cortex**						
R Superior Parietal Lobe	34	-60	50	148	5.52	1.33
L Superior Parietal Lobe	-20	-70	46	†	4.54	0.91
**Ventral Temporal-Occipital Cortex**						
R Fusiform Gyrus/Lingual Gyrus	48	-52	-24	†	6.60	1.83
L Visual Cortex/Fusiform Gyrus/LOC	-10	-68	6	†	6.50	1.06
**Lateral and Medial Temporal Lobe**						
L MTG/Hippocampus/Amygdala	-50	-44	-8	†	5.00	1.03
Post > Pre						
No significant voxels						

Abbreviations: DLPFC = Dorsolateral Prefrontal Cortex; VLPFC = Ventrolateral Prefrontal Cortex; MFG = Middle Frontal Gyrus; SFG = Superior Frontal Gyrus; OFC = Orbitofrontal Cortex; LOC = Lateral Occipital Cortex; MTG = Middle Temporal Gyrus. Note: Height threshold P < .01, corrected for multiple comparisons using Monte Carlo Simulation at P < .01 extent. Effect sizes for group differences are shown as Cohen's d.

†*These are anatomically distinct sub-peaks of the same activated cluster.*

Supplementary Table 7. Tutoring-related decreases in brain activation in the MLD group. Brain regions that showed significant decreases in activation during arithmetic problem solving in the MLD group after tutoring, compared to before tutoring.

Supplementary Methods

Participants

Exclusion Criteria

A total of sixteen children were excluded from the study because they did not meet inclusion criteria for (i) in-scanner motion parameters (total frames interpolated < 20%)[1-7] and adequate whole-brain coverage; (ii) in-scanner performance accuracy (> 50%); and (iii) neuropsychological scores. Specifically, a total of three children with mathematical learning disabilities (MLDs) failed to meet criterion (i); two children (TDs) did not meet criterion (ii) due to response bias in the scanner (i.e. failure understanding the instructions, button box confusions, or missed responses); the rest of the subjects (eleven TDs) were excluded on criterion (iii). Specifically, these included one subject with Full Scale IQ < 80; five subjects whose standard math scores were between 85 and 90 — thus, not meeting criteria for MLD or TD (*see below)*. Five more children were excluded in order to match the groups on reading and IQ measures.

MLD Categorization

MLD status was determined using a *normed-based cut-off criterion*, such that MLDs exhibited markedly below-age-level scores on the Wechsler Individual Achievement Test, Second Edition (WIAT-II)[8]. Specifically, children who scored at or below 85 (i.e. the 16th percentile) on the *Numerical Operations* subtest of the WIAT-II were included in the MLD group, while children whose scores were at or above 90 (i.e. the 25th percentile) on the same test formed the TD group (**Supplementary Table 1**). MLD status was confirmed by a *discrepancy criterion*, such that the MLD group had significantly lower scores on the *Numerical Operations* and *Math Reasoning* subtests of the WIAT-II than expected by their Full-scale IQ (FSIQ), as measured by the Wechsler Abbreviated Scale of Intelligence

(WASI)[9] (**Supplementary Table 2**). Specifically, children with MLD exhibited a significant discrepancy (both $P < .00001$) between their mathematical scores and their FSIQ scores (**Supplementary Table 2**). This discrepancy was not evident for any of the reading measures (all $P > .42$) (**Supplementary Table 2**). Furthermore, there was no significant discrepancy in either math or reading measures in the TD group (all $P > .66$) (**Supplementary Table 2**). The MLD and TD groups did not differ in age, IQ, reading and working memory abilities (**Supplementary Table 1**). Additionally, MLD and TD groups did not differ on measures of math anxiety before ($P > .16$) or after ($P > .53$) tutoring.

Tutoring sessions

The 8-week math tutoring was adapted from MathWise[10-12], and combined conceptual instruction with speeded retrieval of arithmetical facts.

As in MathWise, each lesson was divided into two segments: (*i*) the first part of the lesson was designed to improve number knowledge and (*ii*) the second one was focused on strategic speeded practice. As the child progressed through the tutoring (from lesson 1 to lesson 22), the total time spent on each segment gradually shifted from (*i*) conceptual to (*ii*) procedural.

Lessons 1 through 4 focused on familiarizing the child with math manipulatives (i.e. number line and blocks) and reviewed the operations of addition and subtraction with simple operands such as 0, 1 and 2, as well as small tie problems (from 1 + 1 to 6 + 6 and their corresponding subtraction facts such as 12 - 6). These lessons also had a conceptual component focused on math procedures: they reviewed the commutative property of addition (i.e. changing the order of the operands does not change the resulting sum), as

well as the additive identity property of zero (i.e. adding zero as an operand does not change the number's value). Lessons 5 and 6 taught the "min strategy" for addition (i.e. start counting from the larger number and count up with the smaller number), and the "missing addend strategy" for subtraction (i.e. start with the smaller number and count up to the larger number). During lessons 7 to 22, children practiced these strategies with progressively more difficult problems. They started out with all addition problems that summed to 5, and the corresponding subtraction problems (i.e. "Number Family" of 5). At the end of the program, they had learned all the addition problems that summed to 18, and their corresponding subtraction problems.

Each lesson (**Fig. 1a**) followed the same structure: (i) warm-up physical flashcards to review previously trained math problems; (ii) number knowledge review, including the use of manipulatives and counting strategies; (iii) a lesson worksheet that introduced the new math problems (i.e. the new 'Number Families'); (iv) a physical math game (**Fig. 1b**); (v) computerized (untimed) and physical (timed) flashcards combining both current and previous lessons' material; and (vi) a review worksheet for the 'Number family' covered that day.

Since scanning occurred only on weekends, children who completed lesson 22 early in the week took part in additional review sessions (up to three). Such scenario occurred equally in each group (Fisher's Exact test *P-value* = .22).

Tutoring material

As in MathWise[10-12], the tutoring addressed 200 number combinations with addends and subtrahends from 0 to 9. The conceptual part of the tutoring included physical

manipulatives and a 1 through 19 number line; the tutor and child generated stories of addition and subtraction concepts. Namely, the tutor and child used manipulatives to explore how the target number (e.g. 5 from the "Number Family" of 5) can be partitioned in different ways to derive the adding and subtracting problems comprising that set/number family. They also focused on part-whole knowledge (i.e. problems using the same triplet of numbers – e.g. 2+3 = 5; 3+2 = 5; 5-2 = 3; and 5-3 = 2). Moreover, the child was also asked to generate all addition and subtraction problems (with answers) in that particular target set/number family (e.g. 5), while using the aforementioned manipulatives to represent the problems. Finally, the student reviewed previous sets, orally stating answers to problems with corrective feedback.

During the strategic speeded practice part of the tutoring, the tutor required the child to know the answer (i.e. retrieve it from memory, if confident, or use the efficient counting strategies they had been taught). Here, a series of physical math game were used. These alternated between *Math Bingo* — in which the child has to quickly calculate the sum of a given problem (i.e. 6 + 4) and verify whether the answer (i.e. 10) appears on his/her bingo card (**Fig. 1b**); *Math War* — in which the child and the tutor each draw two cards from a given deck of cards picturing the digits 0 to 9, and quickly calculate the sum of the digits to hopefully end up with the greatest sum (**Fig. 1b**); and *Treasure Hunt* — in which the child has to draw on a deck of cards picturing math problems, solve the given problem on each card and write down the equation as well as the correct answer on the stepping stones of the 'treasure map' provided (**Fig. 1b**).

Children also completed two sets of "flash-cards" activities: (*i*) a computerized, untimed flashcard game, during which a subset of "Number Family" problems — from that lesson

or previous ones — was presented on the screen, the child had to solve them at their own pace, and type in their answer. If the answer were correct, the program acknowledged it and carried on to the next problem, if not, the program displayed the equation together with the correct answer on the screen; (*ii*) a physical, timed flashcard game called "Meet or Beat Your Score", during which the child had 90 seconds to answer a stack of flash cards — i.e. all sets of the "Number Family" problems covered during that lesson. Children could not proceed to the next item until a correct response was generated. If an error occurred in the computation, the tutor encouraged the child to use the most efficient counting strategy to produce the correct response. In the meantime, the 90 seconds continued to elapse as the child used the counting procedure. In this way, correct but quick responses were emphasized.

Functional MRI tasks

Functional Runs' selection

Each child completed at least two functional runs of addition and control problems; in some cases, due to excessive movement, up to four extra runs were acquired. Post-hoc run selection was based on the following criteria: total frames interpolated < 20% and performance accuracy > 50%. For each participant, the final analyses were performed on the first two available runs with the lowest movement meeting the behavioral cut-off criterion (> 50% accuracy). The frequency distribution of selected runs did not differ between groups for either scan session: pre-tutoring (Fisher's exact test *P-value* = .42), post-tutoring (Fisher's exact test *P-value* = .43). Moreover, the distribution of selected runs did not differ within each group over pre- and post- scan sessions. Specifically, in the MLD group (Fisher's exact test *P-value* = .61), and in the TD group (Fisher's exact test *P-value* = .26).

Monte Carlo Procedures Used to Correct for Family-Wise Error Rate

We used a non-parametric approach based on Monte Carlo simulations to determine the minimum cluster size that controls for false positive rate at $P < .01$ for height and $P < .01$ for cluster extent. Monte Carlo simulations were implemented in MATLAB using methods similar to the AlphaSim procedure in the software package Analysis for Functional NeuroImages in 3D (AFNI)[13-15]. Ten thousand iterations of random 3D images, with the same resolution and dimensions as the fMRI data, were generated. The resulting images were masked for gray matter and then smoothed with the same 6mm full-width half-maximum Gaussian kernel used to smooth the fMRI data. The maximum cluster size was then computed for each iteration and the probability distribution was estimated across the 10,000 iterations. The cluster threshold corresponding to a family-wise error significance level of height $P < .01$ and cluster extent $P < .01$ was determined to be 128 voxels. The cluster threshold corresponding to a family-wise error significance level of height $P < .05$ and cluster extent $P < .01$ was determined to be 562 voxels (this applies to **Supplementary Fig. 2** only).

Bayesian analyses

We used Bayesian estimation procedures to validate our findings of null group-differences in the post-tutoring session[16]. We used procedures that provide complete posterior probability distributions of credible values for group means and their differences, as well as robust handling of outliers. These analyses were conducted using the Bayesian Estimation Supersedes the *t*-test (BEST) package implemented in R[16], on the mean *beta* parameter estimates from the General Linear Model (GLM) analysis for our main contrast of interest - *Addition* correct and *Control* correct, using data from all brain regions that

displayed significant differences between MLD and TD children prior to tutoring (**Supplementary Table 3**).

Supplementary References

1 Cho, S. *et al.* Hippocampal-prefrontal engagement and dynamic causal interactions in the maturation of children's fact retrieval. *J. Cogn. Neurosci.* **24**, 1849-1866, doi:10.1162/jocn_a_00246 (2012).

2 Cho, S., Ryali, S., Geary, D. C. & Menon, V. How does a child solve 7 + 8? Decoding brain activity patterns associated with counting and retrieval strategies. *Developmental Sci.* **14**, 989-1001, doi:10.1111/j.1467-7687.2011.01055.x (2011).

3 Redcay, E. *et al.* Atypical brain activation patterns during a face-to-face joint attention game in adults with autism spectrum disorder. *Hum. Brain Mapp.* **34**, 2511-2523, doi:10.1002/hbm.22086 (2013).

4 Romens, S. E. *et al.* Adolescent girls' neural response to reward mediates the relation between childhood financial disadvantage and depression. *J. Child Psychol. Psyc.*, doi:10.1111/jcpp.12410 (2015).

5 Stosic, M., Brass, M., Van Hoeck, N., Ma, N. & Van Overwalle, F. Brain activation related to the perception of minimal agency cues: the role of the mirror system. *Neuroimage* **86**, 364-369, doi:10.1016/j.neuroimage.2013.10.007 (2014).

6 Wendelken, C., O'Hare, E. D., Whitaker, K. J., Ferrer, E. & Bunge, S. A. Increased functional selectivity over development in rostrolateral prefrontal cortex. *J. Neurosci.* **31**, 17260-17268, doi:10.1523/jneurosci.1193-10.2011 (2011).

7 Young, C. B., Wu, S. S. & Menon, V. The neurodevelopmental basis of math anxiety. *Psychol. Sci.* **23**, 492-501, doi:10.1177/0956797611429134 (2012).

8 Wechsler, D. *The Wechsler Individual Achievement Test — Second Edition (WIAT-II).* (San Antonio, TX: The Psychological Corporation, 2001).

9 Wechsler, D. *Wechsler Abbreviated Scale of Intelligence (WASI).* (San Antonio, TX: The Psychological Corporation, 1999).

10 Fuchs, L. S., Powell, S.R., Hamlett, C.L., Fuchs, D., Cirino, P.T., & Fletcher, J.M. . Remediating computational deficits at third grade: a randomized field trial. *J. Res. Educ. Eff.* **1**, 2-32 (2008).

11 Fuchs, L. S. *et al.* Effects of first-grade number knowledge tutoring with contrasting forms of practice. *J. Educ. Psychol.* (2013).

12 Powell, S. R., Fuchs, L.S., Fuchs, D., Cirino, P.T., & Fletcher, J.M. Effects of fact retrieval tutoring on third-grade students with math difficulties with and without reading difficulties. *Learn. Disabil. Res. Pract.: a publication of the Division for Learning Disabilities, Council of Exceptional Children* **24**, 1-11 (2009).

13 Cox, R. W. AFNI: software for analysis and visualization of functional magnetic resonance neuroimages. *Comput. Biomed. Res.* **29**, 162-173 (1996).

14 Nichols T, H. S. Controlling for family-wise error rate in functional neuroimaging: a comparative review. *Stat. Methods Med. Res.* **29**, 162-173 (2003).

15 Xiong J, G. J.-H., Lancaster JL, & Fox PT Clustered pixels analysis for functional MRI activation studies of the human brain. *Hum. Brain Mapp.* **3**, 287-301 (1995).

16 Kruschke, J. K. Bayesian estimation supersedes the t test. *J. Exp. Psychol. Gen.* **142**, 573-603, doi:10.1037/a0029146 (2013).
